# Acute Effect of Alcohol Intake on Cardiovascular Autonomic Regulation During the First Hours of Sleep in a Large Real-World Sample of Finnish Employees: Observational Study

**DOI:** 10.2196/mental.9519

**Published:** 2018-03-16

**Authors:** Julia Pietilä, Elina Helander, Ilkka Korhonen, Tero Myllymäki, Urho M Kujala, Harri Lindholm

**Affiliations:** ^1^ Faculty of Biomedical Sciences and Engineering BioMediTech Institute Tampere University of Technology Tampere Finland; ^2^ Firstbeat Technologies Jyväskylä Finland; ^3^ Department of Psychology University of Jyväskylä Jyväskylä Finland; ^4^ Faculty of Sport and Health Sciences University of Jyväskylä Jyväskylä Finland; ^5^ Finnish Institute of Occupational Health Helsinki Finland; ^6^ Nokia Technologies Espoo Finland

**Keywords:** heart rate, heart rate variability, sleep, alcohol drinking, autonomic nervous system, wearable electronic device

## Abstract

**Background:**

Sleep is fundamental for good health, and poor sleep has been associated with negative health outcomes. Alcohol consumption is a universal health behavior associated with poor sleep. In controlled laboratory studies, alcohol intake has been shown to alter physiology and disturb sleep homeostasis and architecture. The association between acute alcohol intake and physiological changes has not yet been studied in noncontrolled real-world settings.

**Objective:**

The aim of this study was to assess the effects of alcohol intake on the autonomic nervous system (ANS) during sleep in a large noncontrolled sample of Finnish employees.

**Methods:**

From a larger cohort, this study included 4098 subjects (55.81%, 2287/4098 females; mean age 45.1 years) who had continuous beat-to-beat R-R interval recordings of good quality for at least 1 day with and for at least 1 day without alcohol intake. The participants underwent continuous beat-to-beat R-R interval recording during their normal everyday life and self-reported their alcohol intake as doses for each day. Heart rate (HR), HR variability (HRV), and HRV-derived indices of physiological state from the first 3 hours of sleep were used as outcomes. Within-subject analyses were conducted in a repeated measures manner by studying the differences in the outcomes between each participant’s days with and without alcohol intake. For repeated measures two-way analysis of variance, the participants were divided into three groups: low (≤0.25 g/kg), moderate (>0.25-0.75 g/kg), and high (>0.75 g/kg) intake of pure alcohol. Moreover, linear models studied the differences in outcomes with respect to the amount of alcohol intake and the participant’s background parameters (age; gender; body mass index, BMI; physical activity, PA; and baseline sleep HR).

**Results:**

Alcohol intake was dose-dependently associated with increased sympathetic regulation, decreased parasympathetic regulation, and insufficient recovery. In addition to moderate and high alcohol doses, the intraindividual effects of alcohol intake on the ANS regulation were observed also with low alcohol intake (all *P*<.001). For example, HRV-derived physiological recovery state decreased on average by 9.3, 24.0, and 39.2 percentage units with low, moderate, and high alcohol intake, respectively. The effects of alcohol in suppressing recovery were similar for both genders and for physically active and sedentary subjects but stronger among young than older subjects and for participants with lower baseline sleep HR than with higher baseline sleep HR.

**Conclusions:**

Alcohol intake disturbs cardiovascular relaxation during sleep in a dose-dependent manner in both genders. Regular PA or young age do not protect from these effects of alcohol. In health promotion, wearable HR monitoring and HRV-based analysis of recovery might be used to demonstrate the effects of alcohol on sleep on an individual level.

## Introduction

### Background

Sleep is a crucial period of physiological restoration, and it is the optimal state to assess the tonic component or the most relaxed state of the autonomic nervous system (ANS) in real-life conditions [1]. Poor sleep attenuates relaxation in the ANS [2], impairs regenerative physiological processes, causes metabolic disturbances, and has been associated with negative health outcomes [3]. Alcohol intake disturbs recovery, sleep homeostasis, and sleep architecture in several ways [4]. Alcohol affects negatively on stress-related cardiovascular adaptation in the ANS and hypothalamus-pituitary-adrenal axis [5]. Still, alcohol is used to relieve stress [6] or as sleep medicine [4]. Increased alcohol consumption is associated with long working hours, poor social support, and low job control [7].

Heart rate variability (HRV) is a widely used marker of cardiac autonomic regulation reflecting fluctuations in R-R intervals in short or extended time recordings [8] and is modulated by respiration, central vasoregulatory centers, peripheral baroreflex loops, and genetic factors [9]. HRV decreases with age, although differently in men and women [8]. In addition, suppressed HRV has been shown to predict occurrence of different diseases and conditions such as diabetic neuropathy or left ventricular dysfunction after acute myocardial infarction [10]. Traditionally, the HRV analysis is performed in time or frequency domains [10] but also novel analysis methods exist [11]. A widely used time domain measure of HRV is the root mean square of the successive differences (RMSSD) between adjacent R-R intervals, which mainly reflects the parasympathetic input of cardiac regulation [10]. In the frequency domain analysis, the high frequency (HF) band of HRV is considered to indicate parasympathetic regulation [10], whereas the low frequency (LF) band reflects both parasympathetic and sympathetic regulation [10,12]. The ratio between LF power and HF power (LF/HF ratio) has been suggested to reflect the balance between the two branches of the ANS, but this suggestion has not received a consensus [10,12]. Recently, a standardized reporting system in HRV-related behavioral studies was proposed [13].

In addition to the cardiac autonomic regulation, HRV analysis may also provide useful information on sleep [14], the sleeping brain [15], and stress-related insufficient recovery [16], even though autonomic regulation during sleep is complex and varies during different sleep stages [14]. During slow wave sleep (so-called deep sleep), the parasympathetic regulation has been reported to be dominating and the sympathetic regulation to be attenuated, whereas the opposite is true for rapid eye movement sleep [14]. The sufficient amount of slow wave sleep has been associated with good physical and mental recovery [1]. Unconscious stress may be detected in physiological recordings made during cardiovascular stress recovery [17], and HRV may usefully reflect the adaptive resources of the ANS [18]. However, the limitations and pitfalls of HRV analysis as well as the physiological nature of HRV have to be taken into account in all interpretations.

### Prior Work

The effect of acute alcohol intake on the ANS using heart rate (HR) and HRV parameters has been shown in the previous studies. In laboratory settings, high acute alcohol consumption (0.7 g/kg-1.0 g/kg) was associated with decreased HRV and increased HR in awake subjects [19]. The effect was also observed with lower doses (two drinks, not reported in g/kg units) [20]. In one laboratory study, both HRV and polysomnography were monitored after alcohol consumption [21]. The young healthy male subjects (n=10) were given no (0 g/kg, control), low (0.5 g/kg of ethanol), or high (1.0 g/kg) dose of alcohol. A dose-related effect of alcohol on HR and HRV during sleep was found, and the highest HR and lowest HRV were observed for high dose.

However, the effect of acute alcohol intake on the ANS during sleep has not been studied in noncontrolled free-living conditions or with large samples. Most published studies considering the effects of acute alcohol intake on HRV have involved only males, been rather small in number of participants, and included no comparison between genders or objective measurements of physical activity (PA) and recordings during sleep [9]. Thus, studies employing larger number of participants with both genders and considering the background parameters of the subjects such as age, body mass index (BMI) and PA, are needed.

### Goal of This Study

The widespread use of wearable and connected consumer devices enables unobtrusive collection of massive amounts of data from large number of individuals during their daily life. These health-related datasets gathered under normal day-to-day circumstances outside of traditional clinical trials represent so called real-world data [22]. This real-world data collected in uncontrolled settings and outside of clinical trials may be exploited in research to complement the knowledge gained from the traditional clinical trials [23]. The multitude and variety of individuals and information included in real-world datasets allow studying aspects that cannot be studied to that extent in traditional clinical trials [23]. The real-world data has also the prospect to assess the generalizability of the findings from traditional clinical trials with specific populations and circumstances to broader populations and circumstances [22]. On the other hand, the associations found in real-world data can serve as hypotheses for further clinical trials [22]. To gain valid results from the real-world data, the data characteristics such as the sample bias, missing data, confounding, uncertainties and provenance of the data, must, however, be taken into account in the analysis [24].

Alcohol consumption is a universal health behavior associated with poor sleep [4], but to the authors’ knowledge, there is not yet any study employing real-world data [9]. This study analyzes the effects of alcohol intake on the ANS during sleep in a large free-living population. An observational real-world dataset of continuous beat-to-beat R-R interval recordings and self-reported sleep times and alcohol consumption collected from over 40,000 subjects during their normal everyday life was employed for studying retrospectively the effect of acute alcohol intake on sleep. The intraindividual differences in the HRV during sleep associated with acute alcohol intake were studied from 4098 participants of various ages, BMI ranges, and PA levels in whom data with and without alcohol consumption during previous day was available. The purpose of the study was to assess the generalizability of the previous findings to broader population and to study associations between the characteristics of the subjects and the effects of acute alcohol intake on the HR and HRV parameters during sleep.

## Methods

### Data Collection

The original data sample contained 111,025 measurement days from 42,086 Finnish employees representing a wide range of blue- and white-collar workers in varying size companies. Employees had voluntarily participated in a preventive occupational health care program with the aim of improving their health habits and stress management. The program included a continuous beat-to-beat R-R interval recording for a few days during the participant’s normal life. The R-R interval recordings were performed using Bodyguard (Firstbeat Technologies Ltd, Jyväskylä, Finland) wearable device that was attached on the chest with two electrodes. HRV indices, stress, recovery, and PA were computed with Firstbeat Analysis Server (Firstbeat Technologies Ltd) from the recorded R-R interval data, and together with other physiological measurements, they were used as health promotion tools at employees’ workplaces. Employees were instructed not to participate in recordings if they had any disease stages or medications possibly affecting R-R intervals, for example, chronic heart rhythm disturbance, very high blood pressure (≥180/100 mm Hg), type 1 or 2 diabetes with autonomic neuropathy, severe neurological disease (eg, advanced multiple sclerosis or Parkinson disease), fever or other acute disease, or BMI >40 kg/m^2^[25].

All the R-R interval recordings performed on the employees were analyzed and stored anonymously to a registry administered by Firstbeat Technologies Ltd. Each service provider conducting recordings for participants signed an agreement allowing Firstbeat Technologies Ltd to store the anonymized data and to use it for development and research purposes. The employers were responsible to inform their employees about the data usage. Following the agreements, a dataset was extracted from the registry for this study. The use of the dataset for research purposes was approved by the ethics committee of Tampere University Hospital (Reference No R13160).

### Data Extraction

The dataset extracted from the registry to this study included the R-R interval recordings performed with the Bodyguard device (Firstbeat Ltd, Jyväskylä, Finland). The sampling frequency of the device is 1000 Hz for the R-R interval recording [26], and its mean absolute error for R-R intervals has been reported to be 4.45 ms [27]. An artifact correction was performed for the R-R intervals with Firstbeat Analysis Server [28], after which the mean absolute error of R-R intervals has been reported to be 2.27 ms [27]. For this study, the artifact-corrected beat-to-beat R-R intervals were analyzed for a 3-hour period starting 30 min after the self-reported onset of bedtime that is the most likely period for slow wave sleep.

From the artifact-corrected beat-to-beat R-R intervals, the average of HR in 10-min nonoverlapping windows and RMSSD with 5-min windows were calculated [10]. The frequency bands of HRV were assessed applying short-time Fourier transform on the artifact-corrected beat-to-beat R-R interval data. In addition to the traditional HRV measures, personalized HRV-derived indices of recovery were calculated with Firstbeat Analysis Server. The software detects the periods of recovery and thereafter estimates the magnitude of these recovery reactions based on a person’s range of physiological reactions (eg, minimal and maximal HR) and time series variables related to parasympathetic and sympathetic modulation (eg, HR, HF power, LF power, and HRV-derived respiration rate) [29]. During recovery reactions, parasympathetic regulation predominates in the ANS [29]. The momentary absolute level of recovery reactions is estimated with parameters describing the magnitude of parasympathetic modulation, and it is high when HR is individually low and parasympathetic HRV is individually high [30].

**Figure 1:**
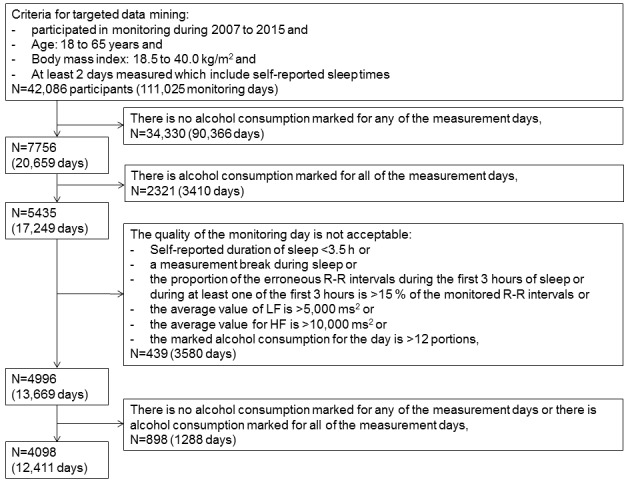
The selection of study population for the analyses.

For this study, the exclusion criteria were unknown or very high reported alcohol consumption (>12 portions of alcohol) during the recording day, unknown or very short self-reported sleeping time, and poor quality of HRV recordings (Figure 1). If a subject reported more than one sleep periods per day, only the longest sleep period was analyzed. Only subjects having a day with at least one portion of alcohol and a day with no alcohol intake were analyzed. The final analysis included 12,411 HRV recording days from 4098 individuals.

As background information, age, gender, and self-reported weight, height, and PA class modified from Ross and Jackson [31] were available. Participants were asked to note their alcohol intake as portions (1 portion=12 g of ethanol) for each measurement day preceding sleep. The exact timing of alcohol intake and smoking history were not available.

### Statistical Analyses

HR, RMSSD, LF/HF ratio, time considered as recovery (recovery percentage), and average of the momentary absolute levels of recovery reactions (recovery index) from a 3-hour period starting 30 min after the self-reported bedtime onset were considered as the outcome variables. Only the first 3 hours of sleep were analyzed, as most of the slow wave sleep typically occurs during the first hours of sleep [1]. During slow wave sleep, the parasympathetic regulation is dominating, and sufficient amount of slow wave sleep has been associated with both good physical and mental recovery [1].

All analyses were conducted in a within-subject repeated-measures manner by comparing the participants’ outcome variables between days with and without alcohol intake. The within-subject design was used, as it allows studying the intraindividual effects of acute alcohol intake and controls for possible unknown confounders.

For the within-subject repeated-measures two-way analysis of variance (ANOVA), the participants’ hourly averages of outcome variables were calculated for days with and without alcohol intake, and the participants were categorized into low (≤0.25 g/kg), moderate (>0.25-0.75 g/kg), and high (>0.75 g/kg) dose groups according to their alcohol intake during the day. Note that the groups also include the participant’s reference with no alcohol, and the participants may have data in one, two, or all three dose groups. If a participant had more than 1 day with low, moderate, or high or with no alcohol intake, the outcome variables were averaged over those days. A repeated-measures two-way ANOVA was performed separately for each dose group to evaluate the difference and the shape of the hour-by-hour pattern in the outcome variables between the days with and without alcohol intake.

In the second analysis, the linear regression model was fitted for the difference in the average of the 3-hour HR and HRV parameters between the participant’s days with and without alcohol intake. First, the 3-hour averages of the outcome variables were calculated for each measurement day. Thereafter, the difference in the participant’s averages between the days with and without alcohol intake was calculated. If the participant had more than one measurement day without alcohol intake, the average of the measurement days’ 3-hour outcome variable averages was employed. A dataset including the measurement day with the highest amount of reported alcohol intake from each participant was extracted, and a linear regression was fitted to the data using the difference in the outcome variables between the days with and without alcohol intake as a dependent variable. In addition to alcohol intake, all information available about the subjects was employed as independent variables in the regression models. The independent variables were continuous variables of alcohol dose (g/kg), age, PA class, BMI, and the 3-hour average of HR (bpm) during a night after a day without alcohol intake (baseline sleep HR) and gender as a categorical variable. Age, BMI, and the baseline sleep HR were subtracted to baseline levels of 18 years, 18.5 kg/m^2^, and 38 bpm, respectively. In addition, a linear regression with interactions between alcohol doses and other predictors was fitted.

All statistical analyses were conducted using R (The R Foundation for Statistical Computing) version 3.2.2. The level of significance in all analyses was set at <0.05. However, with data of this size, it is more important to focus on effect sizes than *P* values [32].

## Results

### Characteristics of the Study Population

From a larger cohort, this study included 4098 subjects who had continuous beat-to-beat R-R interval recordings of good quality with for at least 1 day with and for at least 1 day without alcohol intake. There was a significant proportion of female subjects in this study (Table 1). On average, the subjects were middle-aged, slightly overweight, and had regular PA 2 to 3 times per week, and the total weekly training amount being approximately 1 hour.

Neither PA class nor BMI differed significantly between the dose groups (*P*>.05, Table 2). High alcohol intake was more common among males (*P*<.001) and young subjects (*P*<.001). Average daily alcohol intake in the low, moderate, and high groups was 0.17, 0.45, and 1.1 g/kg, respectively, with the corresponding average number of reported alcohol portions being 1.1, 2.9, and 7.0 drinks.

### Repeated-Measures Analysis of Variance Analyses

**Figure 2:**
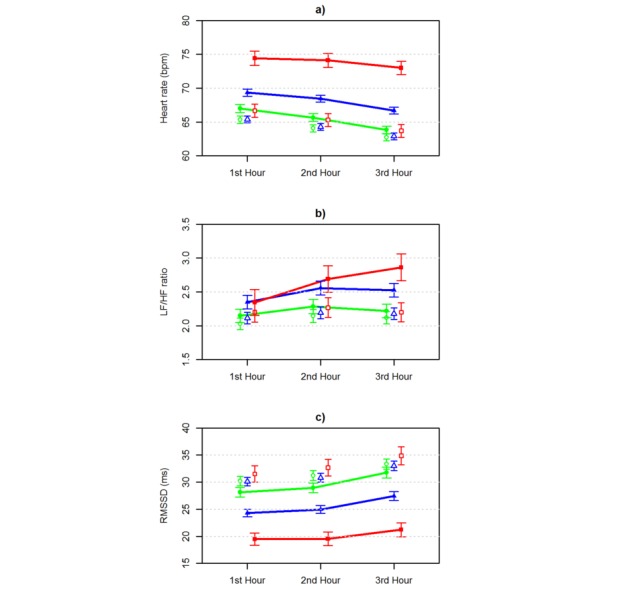
The effect of alcohol intake during the three first hour of sleep on a) heart rate, b) low frequency/high frequency (LF/HF) ratio, and c) root mean square of the successive differences (RMSSD). The marks green ●=low dose (≤0.25 g/kg), blue ▲=medium dose (>0.25-0.75 g/kg), and red ■=high dose (>0.75 g/kg) denote the averages, and corresponding white symbols denote the measures for the same persons without alcohol. Due to the size of the data and clarity of the figure, 99% CIs are shown, and the lines between hours are only shown for alcohol dose groups.

**Figure 3:**
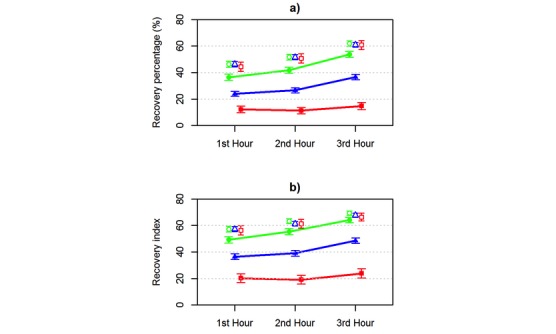
The effect of alcohol intake during the three first hour of sleep on a) recovery percentage, and b) recovery index. The marks green ●=low dose (≤0.25 g/kg), blue ▲=medium dose (>0.25-0.75 g/kg), and red ■=high dose (>0.75 g/kg) denote the averages, and corresponding white symbols denote the measures for the same persons without alcohol. Due to the size of the data and clarity of the figure, 99% CIs are shown, and the lines between hours are only shown for alcohol dose groups.

The means and 99% CIs for HR, the LF/HF ratio, RMSSD, the recovery percentage, and recovery index calculated from intraindividual HRV recordings during the first 3 hours of sleep in low, moderate, and high dose groups were calculated (Figures 2 and 3). Low HR and LF/HF ratio reflect increased parasympathetic regulation, and low RMSSD indicates increased sympathetic regulation in the ANS.

High alcohol intake had the greatest effect on the outcome variables. On average, HR was increased by 1.4 bpm with low, 4.0 bpm with moderate, and 8.7 bpm with high alcohol intake. The LF/HF ratio was increased by 0.1 with low, 0.3 with moderate, and 0.5 with high alcohol intake. RMSSD was decreased by 2.0 ms with low, 5.7 ms with moderate, and 12.9 ms with high alcohol intake. The recovery percentage was decreased by 9.3 percentage units with low, 24.0 percentage units with moderate, and 39.2 percentage units with high alcohol intake. The recovery index was decreased by 7.1 with low, 20.8 with moderate, and 40.2 with high alcohol intake.

For each dose group, the within-subject repeated-measures two-way ANOVA showed significant differences in all outcome variables (all *P*<.001) between the days with and without alcohol intake. In addition, the hourly HRV parameters differed significantly from each other (all *P*<.001). In high dose group comparisons, the interactions between the hour of sleep and alcohol intake were statistically significant for all outcome parameters (all *P*<.001), indicating that the hour-by-hour pattern in the HRV parameters during sleep was different for subjects between the days with high and no alcohol intake. For days with high alcohol intake, the average LF/HF ratio increased hour-by-hour during sleep, whereas the average LF/HF ratio increased from the first to the second hour of sleep but decreased from the second to the third hour of sleep for days with no alcohol intake. For days without alcohol intake, the recovery percentage and recovery index increased as the sleep progressed, but this did not occur after high alcohol intake. In moderate dose group comparisons, the interactions between hour and alcohol intake were statistically significant for the LF/HF ratio (*P*=.002) and recovery percentage (*P*=.01).

**Table 1:** Characteristics of the study population.

Demographic characteristic	All (N=4098), mean (SD; range)	Males (N=1811), mean (SD; range)	Females (N=2287), mean (SD; range)
Age (years)	45.1 (9.6; 19-65)	45.2 (9.4; 19-65)	44.9 (9.8; 19-65)
Physical activity class^a^	4.8 (1.8; 0-10)	4.9 (1.7; 0-10)	4.8 (1.8; 0-10)
Body mass index (kg/m^2^)	26.0 (4.0; 18.5-39.9)	26.7 (3.5; 18.9-39.5)	25.4 (4.3; 18.5-39.9)

^a^Physical activity class range: 0 (physically inactive) to 10 (physically very active).

**Table 2:** Characteristics of low, moderate, and high dose groups during the heart rate variability (HRV) recordings.

Demographic characteristic	Low ≤0.25 g/kg (n=1752)	Moderate >0.25-0.75 g/kg (n=2194)	High >0.75 g/kg (n=716)	*P* value
Number of male subjects, n (%)	671 (38.29)	1010 (46.03)	380 (53.1)	<.001^a^
Age in years, mean (SD)	45.6 (9.0)	46.3 (9.3)	42.3 (10.7)	<.001^b^
Physical activity class, mean (SD)	4.9 (1.6)	4.9 (1.7)	4.6 (1.9)	.59^b^
Body-mass index in kg/m^2^, mean (SD)	25.9 (4.2)	26.0 (3.8)	25.8 (3.7)	.10^b^
Weight in kg, mean (SD)	77.5 (16.2)	77.9 (14.3)	78.1 (14.1)	.31^b^

^a^Chi-square test.

^b^One-way analysis of variance (ANOVA) adjusted for gender.

This shows that the hour-by-hour pattern was different between the days with moderate and no alcohol intake only for the LF/HF ratio and recovery percentage. In low dose comparisons, the hour-by-hour pattern in the LF/HF ratio (*P*=.51), RMSSD (*P*=.06), and recovery percentage (*P*=.08) during sleep was similar between the days with low and no alcohol intake. The HR (*P*<.001) and recovery index (*P*=.01) variables had a statistically significant interaction between the hour and alcohol intake, ie, their hour-by-hour pattern during sleep differed between the days with low and no alcohol intake. Visual inspection (Figures 2 and 3) showed that after low alcohol intake, the levels of outcome variables during the third hour approach their reference levels.

### Linear Models

In linear model analysis, alcohol intake significantly affected the outcome variables (Tables 3 and 4). The results show that HR was increased with acute alcohol intake. For example, alcohol intake of 0.75 g/kg increased HR for subjects on average by 6.8 bpm compared with their nights without alcohol intake (Table 3). Alcohol intake increased the HR significantly more among young than older subjects: alcohol intake of 0.75 g/kg increased HR on average by 1.8 bpm more for a 30-year old person than for a 60-year old person (Table 4). In addition, alcohol intake increased HR significantly more among subjects with lower than higher baseline sleep HR: alcohol intake of 0.75 g/kg and an increase of 10 bpm in baseline HR decreased the difference in HR by 3.4 bpm, on average (Table 4). The increase in HR with alcohol intake was similar for subjects despite their gender, PA level, or BMI (Table 4).

Alcohol intake increased the LF/HF ratio, and this effect was slightly stronger among males and subjects with higher PA level (Table 4). However, the coefficients of determination for the LF/HF ration linear regression models were low (Tables 3 and 4), indicating that the input variables employed in the models did not explain the variation in the LF/HF ratio well. RMSSD was decreased by alcohol intake at all ages, but the effect was stronger in younger than older subjects (Table 4). On average, RMSSD was decreased with high alcohol intake (0.75 g/kg) by 10.9 ms for a 30-year old subject but only by 4.7 ms for a 60-year old subject (Table 4). In addition, alcohol intake decreased RMSSD more for subjects with lower baseline HR (Table 4).

Recovery percentage decreased significantly by increased alcohol intake (Tables 3 and 4). An 80-kg person drinking five portions of alcohol (0.75 g/kg) has on average 45 min less recovery (25.25 percentage units) during the first 3 hours of sleep than without alcohol (Table 3). The recovery percentage was decreased significantly more by alcohol intake for subjects with lower baseline HR than with higher baseline HR (Table 4). The decrease in recovery percentage with alcohol was similar regarding the other background parameters of the subjects (Table 4). In addition, the recovery index was attenuated with alcohol intake (Tables 3 and 4). Alcohol intake attenuated the recovery index slightly more in subjects with higher BMI than with lower BMI (Table 4). The other background parameters did not have a significant interaction with alcohol intake (Table 4).

When the effects for alcohol and background characteristics were controlled, the difference in recovery percentage was strongly correlated with the difference in HR (Pearson partial correlation coefficient and the coefficient of determination: *r*=−.70, *R*^2^=.486, *P*<.001) and in RMSSD (*r*=.51, *R*^2^=.262, *P*<.001) but only moderately correlated with the change in LF/HF ratio (*r*=−.27, *R*^2^=.071, *P*<.001). Similarly, the difference in the recovery index was strongly correlated with the difference in HR (*r*=−.63, *R*^2^=.388, *P*<.001) and in RMSSD (*r*=.49, *R*^2^=.236, *P*<.001) but only moderately correlated with the change in LF/HF ratio (*r*=−.27, *R*^2^=.074, *P*<.001). The partial correlation between the difference in the recovery percentage and the recovery index was moderate (*r*=.48, *R*^2^=.229, *P*<.001).

**Table 3:** The linear regression models without interaction components for the average of heart rate (HR), low frequency/high frequency (LF/HF) ratio, root mean square of the successive differences (RMSSD), recovery percentage, and recovery index during the first 3 hours of sleep. BMI: body mass index.

Outcome	HR	LF/HF ratio	RMSSD	Recovery percentage	Recovery index
Intercept, estimate (SE)	10.87 (0.66)^a^	0.715 (0.115)^a^	−15.32 (0.98)^a^	−56.09 (3.25)^a^	−28.27 (3.70)^a^
Alcohol (g/kg), estimate (SE)	8.49 (0.29)^a^	0.425 (0.051)^a^	−12.24 (0.44)^a^	−33.67 (1.45)^a^	−36.63 (1.65)^a^
Physical activity class, estimate (SE)	−0.48 (0.06)^a^	−0.019 (0.011)	0.37 (0.09)^a^	1.62 (0.31)^a^	1.81 (0.35)^a^
Age (0=18 years), estimate (SE)	−0.03 (0.01)^b^	−0.001 (0.002)^a^	0.14 (0.02)^a^	−0.06 (0.05)	−0.08 (0.06)
BMI (0=18.5 kg/m^2^), estimate (SE)	0.22 (0.03)^a^	0.002 (0.005)	−0.26 (0.04)^a^	−0.81 (0.14)^a^	−0.89 (0.15)^a^
Gender (0=female, 1=male), estimate (SE)	−1.70 (0.21)^a^	0.014 (0.037)	2.09 (0.32)^a^	7.22 (1.06)^a^	5.24 (1.21)^a^
Baseline sleep HR (0=38 bpm), estimate (SE)	−0.33 (0.01)^a^	−0.021 (0.002)^a^	0.41 (0.02)^a^	1.67 (0.06)^a^	0.86 (0.07)^a^
Adjusted coefficient of determination for the model	0.267	0.039	0.245	0.230	0.135

^a^*P*<.001.

^b^*P*<.01.

**Table 4:** The linear regression models with interaction components for the average of heart rate (HR), low frequency/high frequency (LF/HF) ratio, root mean square of the successive differences (RMSSD), recovery percentage, and recovery index during the first 3 hours of sleep.

Outcome	HR	LF/HF ratio	RMSSD	Recovery percentage	Recovery index
Intercept, estimate (SE)	7.61 (1.04)^a^	0.724 (0.182)^a^	−10.55 (1.55)^a^	−49.34 (5.13)^a^	−26.73 (5.86)^a^
Alcohol (g/kg), estimate (SE)	14.47 (1.56)^a^	0.386 (0.272)	−20.68 (2.32)^a^	−46.05 (7.69)^a^	−38.48 (8.78)^a^
Physical activity class, estimate (SE)	−0.33 (0.10)^b^	−0.051 (0.018)^b^	0.51 (0.15)^a^	2.29 (0.51)^a^	2.57 (0.59)^a^
Age (0=18 years), estimate (SE)	0.03 (0.02)	0.004 (0.003)	−0.008 (0.03)	0.07 (0.09)	−0.05 (0.10)
BMI (0=18.5 kg/m^2^), estimate (SE)	0.19 (0.04)^a^	0.005 (0.008)	−0.18 (0.07)^b^	−0.60 (0.22)^b^	−0.42 (0.25)
Gender (0=female, 1=male), estimate (SE)	−1.38 (0.35)^a^	−0.010 (0.062)	2.04 (0.53)^a^	6.47 (1.75)^a^	2.95 (2.00)
Baseline sleep HR (0=38 bpm), estimate (SE)	−0.28 (0.02)^a^	−0.019 (0.004)^a^	0.31 (0.03)^a^	1.25 (0.10)^a^	0.71 (0.11)^a^
Alcohol x physical activity class, estimate (SE)	−0.27 (0.17)	0.065 (0.029)^c^	−0.32 (0.25)	−1.47 (0.83)	−1.59 (0.95)
Alcohol x age	−0.12 (0.03)^a^	−0.009 (0.005)	0.26 (0.04)^a^	−0.04 (0.14)	0.27 (0.16)
Alcohol x BMI, estimate (SE)	0.08 (0.08)	−0.009 (0.014)	−0.15 (0.12)	−0.39 (0.39)	−1.05 (0.45)^c^
Alcohol x gender, estimate (SE)	−0.56 (0.60)	0.251 (0.104)^c^	−0.13 (0.89)	1.35 (2.95)	4.87 (3.37)
Alcohol x baseline sleep HR, estimate (SE)	−0.08 (0.03)^c^	−0.005 (0.005)	0.18 (0.05)^a^	0.83 (0.15)^a^	0.30 (0.17)
Adjusted coefficient of determination for the model	0.271	0.042	0.255	0.236	0.137

^a^*P*<.001.

^b^*P*<.01.

^c^*P*<.05.

## Discussion

### Principal Findings

Impact of alcohol on autonomic nervous system control during sleep has been earlier demonstrated in controlled conditions with relatively small samples. This study demonstrated that this effect is also clearly seen in noncontrolled conditions with wearable HR monitoring and HRV analysis. In the large heterogeneous, noncontrolled, and free-living study population, alcohol intake caused a dose-dependent effect in cardiac autonomic regulation during the first 3 hours of self-reported sleep time. Intraindividually, HR remained elevated, parasympathetic recovery was delayed, and sympathetic dominance was prolonged after alcohol intake compared with recordings with no alcohol. The effects in cardiac autonomic regulation were observed already with low doses of alcohol.

These findings accord with previous studies reporting dose-related effects of alcohol on parasympathetic indices of HRV during sleep in laboratory conditions [21]. Increased HR partly explains the attenuated HRV indices during sleep following alcohol intake [33], and prolonged elevation in the LF/HF ratio supports the role of sympathetic regulation in alcohol-related delayed HRV recovery [34]. Even a moderate amount of alcohol was shown to attenuate recovery in the ANS in this study. This accords with the results of a previous study where two drinks caused significantly decreased RMSSD and increased HR and LF/HF ratio, and one drink altered RMSSD but not HR or LF/HF ratio [20]. In this study, HR and the LF/HF ratio were affected also in the low dose (≤0.25 g/kg) group where about 90% of the measurement days involved one drink and the rest two drinks.

The strength of this study was the large study population representing a sample of Finnish employees with the whole span of working age, different BMI categories and PA levels, and both genders. With the large free-living sample, this study provided real-world evidence and enabled further studying the effects of personal background parameters on the effects of alcohol intake on the ANS. The main limitations of the study were not knowing the exact alcohol doses and the exact times of alcohol consumption and sleep. The higher alcohol intakes may have been underestimated. In addition, the alcohol drinking habits of the participants were not known.

Most previous studies considering the effects of alcohol on the ANS have used male subjects only, and differences between the sexes have not been examined [9]. With a significant proportion of female participants, this study showed alcohol mainly affecting the ANS similar among men and women, although the LF/HF ratio showed sympathetic dominance being slightly stronger in men than in women after alcohol intake. The large age range of the participants allowed studying the interaction effect of age and alcohol intake on the ANS. The effect of alcohol intake on the change in HR and RMSSD was stronger in young subjects than in older subjects, but the effect of alcohol on the LF/HF ratio, the recovery percentage, or the recovery index was not age-dependent.

Our findings on the modifiable disease risk factors are in agreement with previous data on that physical inactivity and high BMI reduce HRV [35] and show that consumption of alcohol reduces HRV in all the PA and BMI categories. In fact, this study showed that regular PA does not attenuate the effects of alcohol intake on the ANS. The changes in HR and RMSSD because of alcohol intake were similar for physically active and sedentary participants in this study. The physically active participants actually displayed in LF/HF ratio even stronger intraindividual sympathetic dominance because of alcohol intake than the sedentary subjects did. Consequently, being physically active does not seem to protect from the negative effects of alcohol intake on the ANS during sleep. This aspect is important to consider given that the alcohol consumption is common also among physically active individuals, and there may even be a dose-response relationship between alcohol consumption and level of PA [36]. Even though exercise is beneficial for general health among alcohol users [37], alcohol has been reported to negatively affect HRV recovery after exercise [38]. Thus, clinically important is to note that the risk of exercise-related cardiac events might be raised by prolonged sympathetic tone during recovery. PA on the current day of alcohol intake, a factor that might affect HRV parameters [9], was not estimated, although it would be possible with our material. However, the effect of alcohol on the amount of recovery during sleep has been reported to strongly overwhelm the effect of other daily activities, including PA [39].

Poor sleep associates with negative health behaviors, ill health, and decreased work ability [40,41]. This study might offer some new tools for health promotion in occupational and primary health care to show practically, on individual and personal level, based on wearable HRV monitoring, the negative effects of alcohol on sleep. Demonstration of the insufficient recovery after using alcohol may be very important for individuals who consume alcohol repeatedly day after day and may suffer from accumulated consequences of insufficient recovery. The personalized indices of recovery, recovery percentage, and recovery index were found to accord with the RMSSD and HR. Importantly, the recovery percentage was found to be independent of age, and the recovery index had only a slight interaction effect between alcohol intake and BMI. These personalized recovery parameters can be used as a tool of health promotion in occupational health care to better manage interindividual differences in HRV and to visualize the associations between alcohol consumption and sleep.

### Conclusions

The study demonstrates, with big uncontrolled data from unobtrusive wearable monitoring, that alcohol intake results in suppression of parasympathetic regulation of the ANS in a dose-response manner. Being physically active and young appears to provide no protection from alcohol-induced suppression of parasympathetic regulation, a finding that needs to be considered given the literature evidence that increased PA associates with higher alcohol usage among nonalcoholics. Personalized HRV measures such as recovery percentage may be more practical in occupational health settings to demonstrate the effect of alcohol on sleep than, eg, RMSSD, which is strongly age-dependent.

## Abbreviations

ANS: Autonomic nervous system
ANOVA: Analysis of variance
BMI: Body mass index
HF: high frequency
LF: low frequency
HR: heart rate
HRV: heart rate variability
PA: physical activity
RMSSD: root mean square of the successive differences
